# Estimating the burden of fungal disease in Vietnam

**DOI:** 10.1111/myc.12382

**Published:** 2015-10-09

**Authors:** J. Beardsley, D. W. Denning, N. V. Chau, N. T. B. Yen, J. A. Crump, J. N. Day

**Affiliations:** ^1^ Oxford University Clinical Research UnitHo Chi Minh CityVietnam; ^2^ The National Aspergillosis CentreThe University of ManchesterManchesterUK; ^3^ Hospital for Tropical DiseasesHo Chi Minh CityVietnam; ^4^ Pham Ngoc Thach HospitalHo Chi Minh CityVietnam; ^5^ Centre for International HealthUniversity of OtagoDunedinNew Zealand; ^6^ Wellcome Trust Intermediate FellowLondonUK

**Keywords:** Burden, epidemiology, fungal, incidence, prevalence, Vietnam

## Abstract

Data regarding the prevalence of fungal infections in Vietnam are limited yet they are likely to occur more frequently as increasingly sophisticated healthcare creates more iatrogenic risk factors. In this study, we sought to estimate baseline incidence and prevalence of selected serious fungal infections for the year 2012. We made estimates with a previously described actuarial method, using reports on the incidence and prevalence of various established risk factors for fungal infections from Vietnam, or similar environments, supplemented by personal communications. Global data were used if local data were unavailable. We estimated 2 352 748 episodes of serious fungal infection occurred in Vietnam in 2012. Frequent conditions included recurrent vaginal candidiasis (3893/100 000 women annually), tinea capitis (457/100 000 annually) and chronic pulmonary aspergillosis (61/100 000/5 year period). We estimated 140 cases of cryptococcal meningitis, 206 of penicilliosis and 608 of *Pneumocystis jirovecii* pneumonia. This is the first summary of Vietnamese fungal infections. The majority of severe disease is due to *Aspergillus* species, driven by the high prevalence of pulmonary tuberculosis. The AIDS epidemic highlights opportunistic infections, such as penicilliosis and cryptococcosis, which may complicate immunosuppressive treatments. These estimates provide a useful indication of disease prevalence to inform future research and resource allocation but should be verified by further epidemiological approaches.

## Introduction

Invasive mycoses are serious, with case fatality ratios up to 70%,^1^ and their incidence is likely to rise in rapidly developing countries (such as Vietnam) as increasing affluence leads to better access to more sophisticated treatments, like prolonged intensive care and different forms of iatrogenic immunosuppression.^2^ An assessment of baseline incidence is vital to facilitate the work of healthcare planners and public health professionals. Estimates suggest that over 90% of fungal infections resulting in death are caused by *Candida*,* Cryptococcus*,* Aspergillus* and *Pneumocystis* – together they cause over two million life‐threatening infections globally, each year.^3^ The incidence of cryptococcal meningitis (CM) and *Pneumocystis jirovecii* pneumonia (PCP) are closely linked to the HIV pandemic,^4^ and much of the burden of *Aspergillus* is bound to pulmonary tuberculosis (PTB),^5^, ^6^ which means that most cases of serious mycoses are likely to occur in areas where HIV and TB are prevalent.^7^

Despite the amount of illness and death resulting from fungal infections, these conditions struggle with a low profile and receive little research funding. It has been estimated that just 1.4–2.5% of the immunology and infection research resources of major funders is allocated to invasive mycoses.^3^ These conditions disproportionately affect countries with limited resources, so the lack of research and maldistribution of treatments^8^ raises issues of research equity.^9^ However, even well‐resourced healthcare settings frequently neglect systematic surveillance of fungal infections.^3^ There is no surveillance programme for fungal infections in Vietnam, and their epidemiology is largely unknown. The volume of data related to penicilliosis^3^, ^10^ and cryptococcosis^11^, ^12^, ^13^ is increasing but accurate national estimates of incidence are absent, and the local data on *Candida*,* Aspergillus* or *Pneumocystis* are even sparser.

National community‐based surveillance programmes are the gold‐standard for estimating disease prevalence and incidence, but they are extremely expensive and difficult to implement. Methods using sentinel surveillance have been described to provide data at a lower cost.^14^, ^15^, ^16^, ^17^ These approaches can become complicated and must be adjusted in the urbanised, densely populated communities found in Asia, where overlapping healthcare providers are the norm,^18^, ^19^ and they still require considerable resources. Several researchers have recently undertaken an actuarial approach to estimating the burden of fungal infections at the national level,^20^, ^21^, ^22^, ^23^ based on an approach most clearly described for aspergillosis.^5^, ^6^

We used this method and local data to estimate the burden of selected serious mycoses in Vietnam; the first attempt of its kind in South East Asia.

## Materials and methods

### Population

Foundational data on population structure and risk factors for fungal infections were first identified. We used 2012 WHO population estimates,^24^ and described the population structure with the 2009 Vietnam census data.^25^ Other data sources are identified under relevant disease headings.

### Local disease and risk factor data

We searched for any reports on the epidemiology of fungal infections in Vietnam to inform this work, using PubMed, with variations in the keywords ‘Vietnam’, ‘incidence’, ‘prevalence’, ‘epidemiology’, ‘condition of interest’. We also searched for local information on the prevalence of risk factors such as HIV/AIDS, chronic lung disease, haematological disease, organ transplantation and intensive care. These were supplemented by personal communications with local colleagues who had previously published in the field. Where local reports were not available, the literature was searched for relevant data from other South‐East Asian countries. If such data were also unavailable, international reports were used as the best available surrogate.

### AIDS defining mycoses

We derived data on the epidemiology of HIV and AIDS, and the coverage of anti‐retroviral therapy (ART), from Vietnamese Ministry of Health, UNAIDS, WHO and Global Burden of Disease 2013 reports.^7^, ^24^, ^26^, ^27^The number of new AIDS diagnoses is estimated each year in Vietnam, but not the type of presentation. On the basis of regional and international reports, we assumed that of new AIDS diagnoses in 2012, 3% would be cryptococcal meningitis,^1^ 13% *Pneumocystis jirovecii* pneumonia^28^, ^29^, ^30^ and 4% penicilliosis.^10^

### Candidal infections

The incidence of candidaemia was considered to occur at a rate of five per 100 000 population per year, based on data from international sources, with 1.5 occurring among intensive care unit (ICU) patients and 3.5 in others.^31^ Half of the infections occurring in ICU patients were considered to result from candidal peritonitis.^32^ We obtained data about the number of ICU beds in 2012 from the General Statistics Office of Vietnam.^25^ We estimated oesophageal candidiasis would occur in 20% of ART naïve HIV patients and in 5% of those already on treatment.^33^, ^34^

### *Aspergillus* infections

Disease related to *Aspergillus* was calculated in four categories – invasive aspergillosis (IA), allergic bronchopulmonary aspergillosis (ABPA), severe asthma with fungal sensitisation (SAFS) and chronic pulmonary aspergillosis (CPA). We applied the following multipliers, derived from data from the French Mycosis Study Group,^35^ to local disease statistics to reach an estimate of IA incidence: 10% of patients with acute myeloid leukaemia (AML) and 10% of patients with non‐AML haematological malignancies,^36^, ^37^ 0.5% of renal transplant patients,^38^ 4% of lung transplant patients,^38^ 6% of heart transplant patients^38^ and 4% of liver transplant patients.^38^ It was further estimated, based on incidence data from China, that IA would occur in 3.9%^39^ of all admissions for chronic obstructive pulmonary disease (COPD).^40^

We estimated ABPA would occur in 2.5% of asthmatics,^5^ and the local prevalence of asthma was sourced from a global survey.^41^ No multiplier was applied for cases of cystic fibrosis (CF), as has been done in other national estimates, because cases of CF are negligible in Vietnam. Prevalence of SAFS was calculated as being 33% of the most severe 10% of asthmatics.^5^, ^41^

The method for deriving the prevalence of CPA has been described elsewhere,^5^, ^6^ with the main modification for Vietnam being that cavities occur in 40.8% of pulmonary TB cases.^42^, ^43^ In brief, we established the annual incidence of TB from 2012 WHO estimates,^24^ then estimated that 22% of those with cavities and 2% of those without would develop CPA. We subtracted annual expected deaths amongst those survivors, over 5 years, repeat to establish a 5‐year post‐TB period prevalence. Based on Asian data, a conservative estimate that 75% of CPA cases result from TB was made to facilitate estimation of non‐TB cases.^44^, ^45^

### Other mycoses

We estimated the incidence of mucormycosis to be 1.2 per 1 000 000 population,^46^ fungal keratitis as seven per 100 000 population^47^ and the prevalence of recurrent vulvovaginal candidiasis as 6% of women over 50 years.^48^ The prevalence of tinea capitis has not been described in South East Asia – most international reports of school‐based surveillance give results varying between 0.1% and 9–11%.^49^, ^50^, ^51^ We selected a prevalence of 2% of school‐aged children based on the mean incidence from several surveys in London.^52^

## Results

### Population and country profile

**Figure 1:**
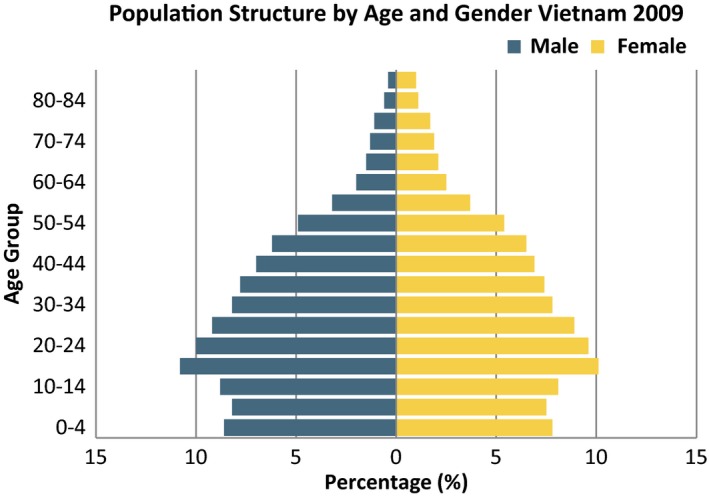
Population structure by age and gender in Vietnam, 2009.

Vietnam is classified as a lower middle income country by the World Bank^53^ and is undergoing pivotal changes in terms of economic development. The population in 2012 was almost 91 000 000 with 23% under 15 years old (20 791 284) and 17% of women over 50 years (6 080 479),^25^ assuming the population structure has changed little since 2009. Figure 1 shows the population structure of Vietnam.

### HIV and respiratory risk factors

In 2012, there were an estimated 250 000 HIV‐infected individuals and 4500 new cases of AIDS – almost half of HIV patients were receiving antiretrovirals (114 900).^26^, ^27^ Vietnam had a high TB burden, with an annual incidence of 218/100 000.^24^ The prevalence of COPD and asthma were 6.7%^40^ and 1%^41^ respectively, which would be expected to result in 348 992 acute admission for COPD.^40^

### Haematological and transplant risk factors

The incidence of acute myeloid leukaemia was approximately five per 100 000 population. There were approximately 20–25 cases of stem cell transplantation [Personal Communication Dr. Huynh Van Man, Transplantation dept, Blood Transfusion and Hematology Hospital in HCMC]. In 2012, 130 kidneys, three hearts and four livers were transplanted,^38^ but with no national registry these figures are approximate. No lung transplant procedures have yet been reported.

### Burden of fungal infections

Table 1 shows the incidence rates and prevalence for selected fungal infections in Vietnam, 2012.

**Table 1:** Total case numbers, prevalence and incidence rates of selected serious fungal infections in Vietnam, 2012

Infection	Estimation method	Total	Cases incidence/prevalence per 100 k population
Cryptococcal meningitis	3% of new AIDS diagnoses	140	0.15
Pneumocystis pneumonia	13% of new AIDS diagnoses	608	0.67
Penicilliosis	4% of new AIDS diagnoses	206	0.23
Candidaemia	5/100 000 general population: 3.5 in ICU patients, 1.5 in non‐ICU patients	4540	5
Oesophageal candidiasis	20% of HIV patients not on ARVs; 5% of those on ARVs	33 107	36
Invasive aspergillosis	3.9% severe COPD; 10% AML; 10% non‐AML haematological malignancy; 0.5% renal transplants; 6% heart transplants; 4% liver transplants	14 523	15.99
Allergic bronchopulmonary aspergillosis (ABPA)	2.5% of adult asthmatics; 15% of adults with cystic fibrosis	23 607	26^a^
Severe asthma with fungal sensitisation (SAFS)	33% of the most severe 10% of adult asthmatics	31 161	34^a^
Chronic pulmonary aspergillosis (CPA)	22% of cases of cavitary pulmonary TB; 2% of non‐cavitary cases	55 509	61^a^
Mucormycosis	1.2 cases per 1 000 000 population	109	0.12
Fungal keratitis	7 cases per 100 000 population	6356	7
Recurrent vaginal candidiasis >4/times/year	6% of women >50 years old	1 767 581	3893^a^
Tinea capitis	2% children <14 years old	415 301	457^a^
Estimated cases		2 352 748	

a Prevalence. Otherwise figures represent annual incidence rate.

We estimate 2 352 748 episodes of serious fungal infection occurred in Vietnam in 2012. The commonest conditions were those associated with lower case fatality ratios, but considerable morbidity, such as recurrent vaginal candidiasis (prevalence of 3893 per 100 000 women) and tinea capitis (prevalence of 457 per 100 000 population). A total of 6356 cases of fungal keratitis were estimated.

Chronic pulmonary aspergillosis had a prevalence of 61 per 100 000. We estimated that there were 4450 cases of candidaemia, 608 of *Pneumocystis jirovecii* pneumonia, 206 of penicilliosis and 140 of cryptococcal meningitis in 2012.

## Conclusions

In Vietnam the major drivers of the most serious fungal infections are the high incidence of TB (leading to *Aspergillus* related disease) and the HIV epidemic (leading to PCP, penicilliosis and CM). Although the prevalence of HIV is not high, the country's large population means there are many individuals at risk. The estimated incidence of candidaemia is also of concern. All of these serious mycoses are associated with high case fatality rates and morbidity. They require long hospital admissions and prolonged courses of anti‐fungal drugs. The health economic implications of these conditions are poorly described in settings such as Vietnam, but are likely to be considerable – cost of disease estimates are urgently required for proper healthcare planning, and to project resource requirements as economic development leads to a rise in iatrogenic risk factors.

Chronic mycoses such as recurrent vulvovaginal candidiasis and tinea capitis are not only inconvenient, but can be stigmatizing – further work is required to delineate the problem and ensure access to therapy is available. The incidence of sight‐threatening fungal keratitis is high; identifying and mitigating risk factors should be a priority.

There are limitations to this actuarial approach to describing the incidence and prevalence of serious mycoses. Some of the calculations are likely to underestimate the true extent of the problem: for example, no attempt has been made to consider the impact of glucocorticosteroid use. The approach has not been fully validated, and certainly not in a tropical setting where mycoses may be more common. Furthermore, without better health economic data it is not possible to make a proper estimate of the burden of disease.

This is, however, the first systematic attempt to describe serious mycoses in Vietnam, or South East Asia, and provides a starting point from which to better understand the extent of the problem. The data presented here should stimulate interest in surveillance of these conditions and will contribute to a growing global effort, co‐ordinated by LIFE (www.LIFE‐worldwide.org), to raise the profile of these neglected conditions.

## Conflict of Interest

JB, NVC, NTBY, JAC, and JND declare no conflict of interest. DWD holds Founder shares in F2G Ltd a University of Manchester spin‐out antifungal discovery company, in Novocyt which markets the Myconostica real‐time molecular assays and has current grant support from the National Institute of Allergy and Infectious Diseases, National Institute of Health Research, NorthWest Lung Centre Charity, Medical Research Council, Astellas and the Fungal Infection Trust. He acts as a consultant to T2 Biosystems, GSK, Sigma Tau, Oxon Epidemiology and Pulmicort. In the last 3 years, he has been paid for talks on behalf of Astellas, Dynamiker, Gilead, Merck and Pfizer. He is also a member of the Infectious Disease Society of America Aspergillosis Guidelines and European Society for Clinical Microbiology and Infectious Diseases Aspergillosis Guidelines groups. He is also President of the Global Action Fund for Fungal Infections.
